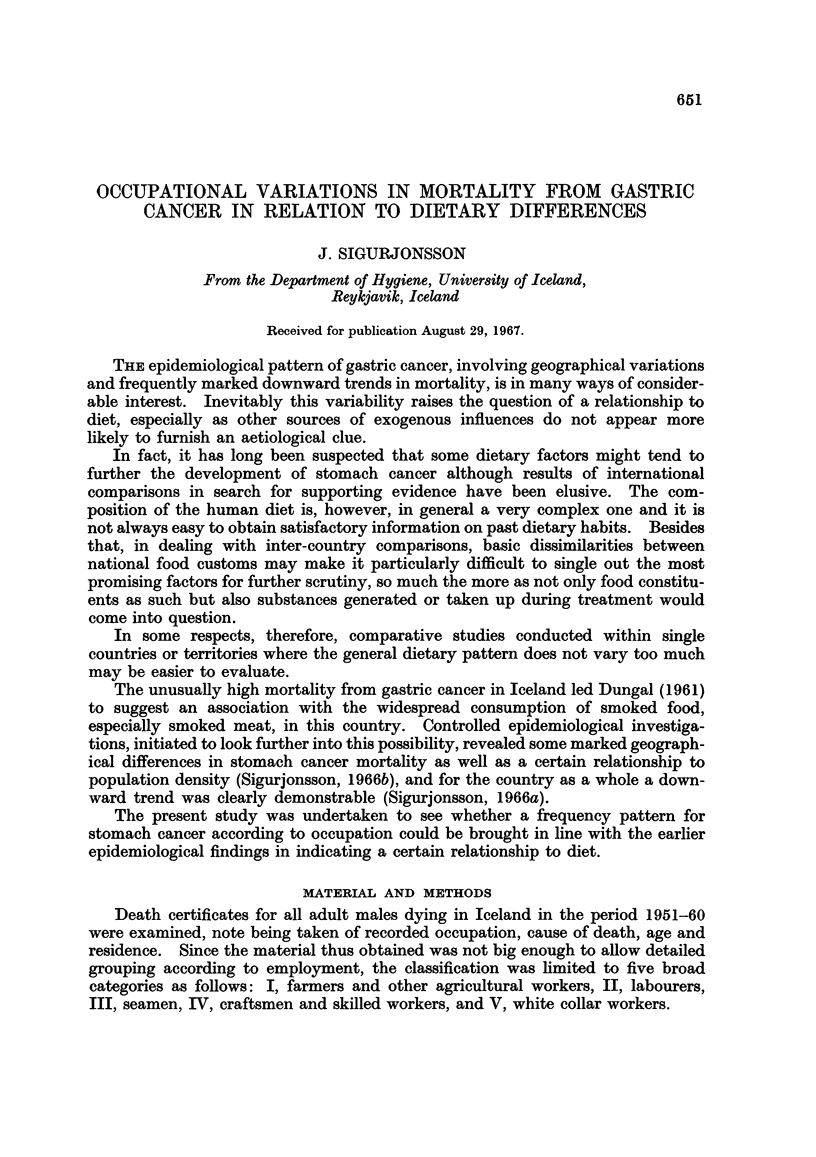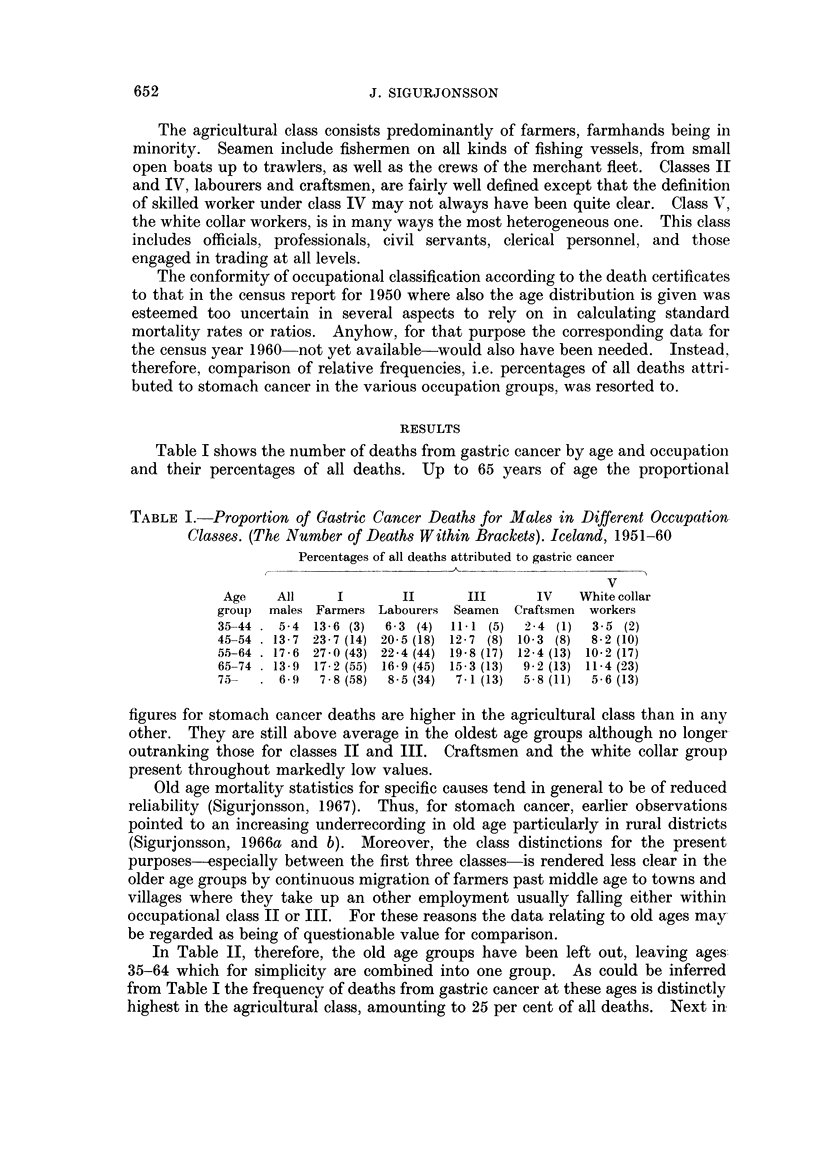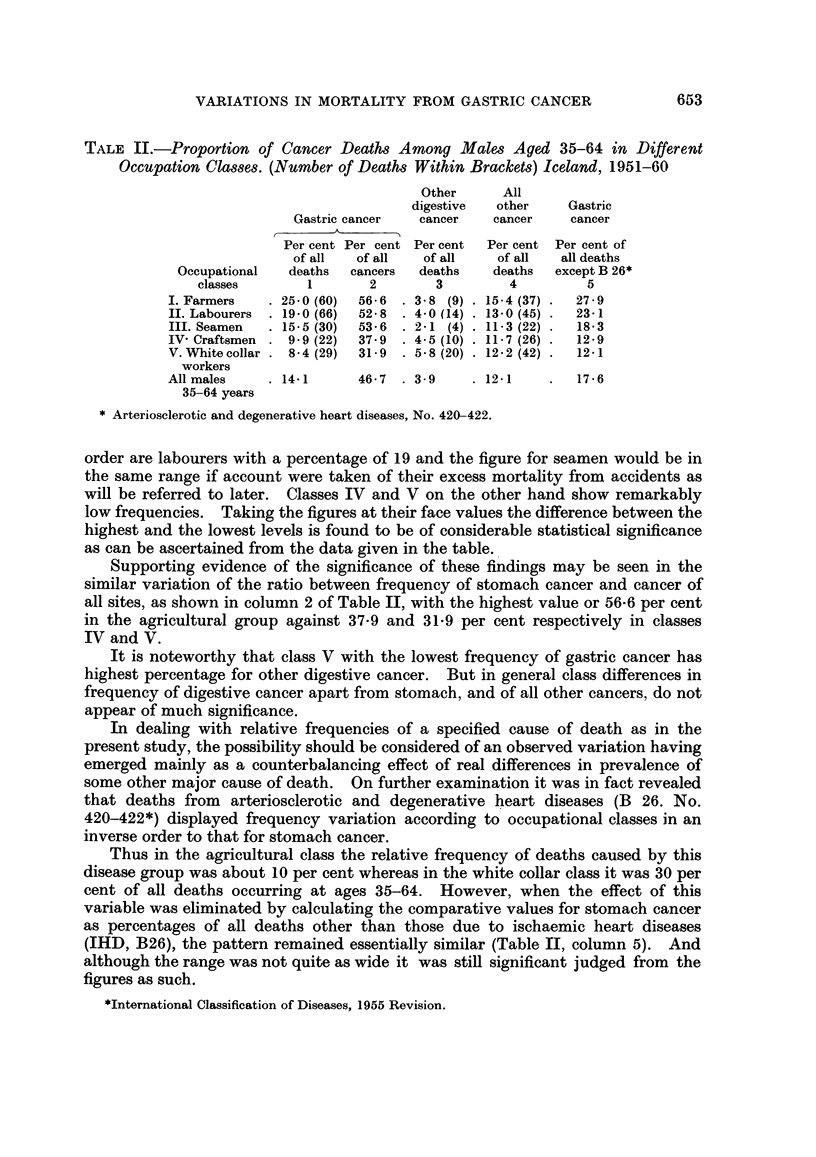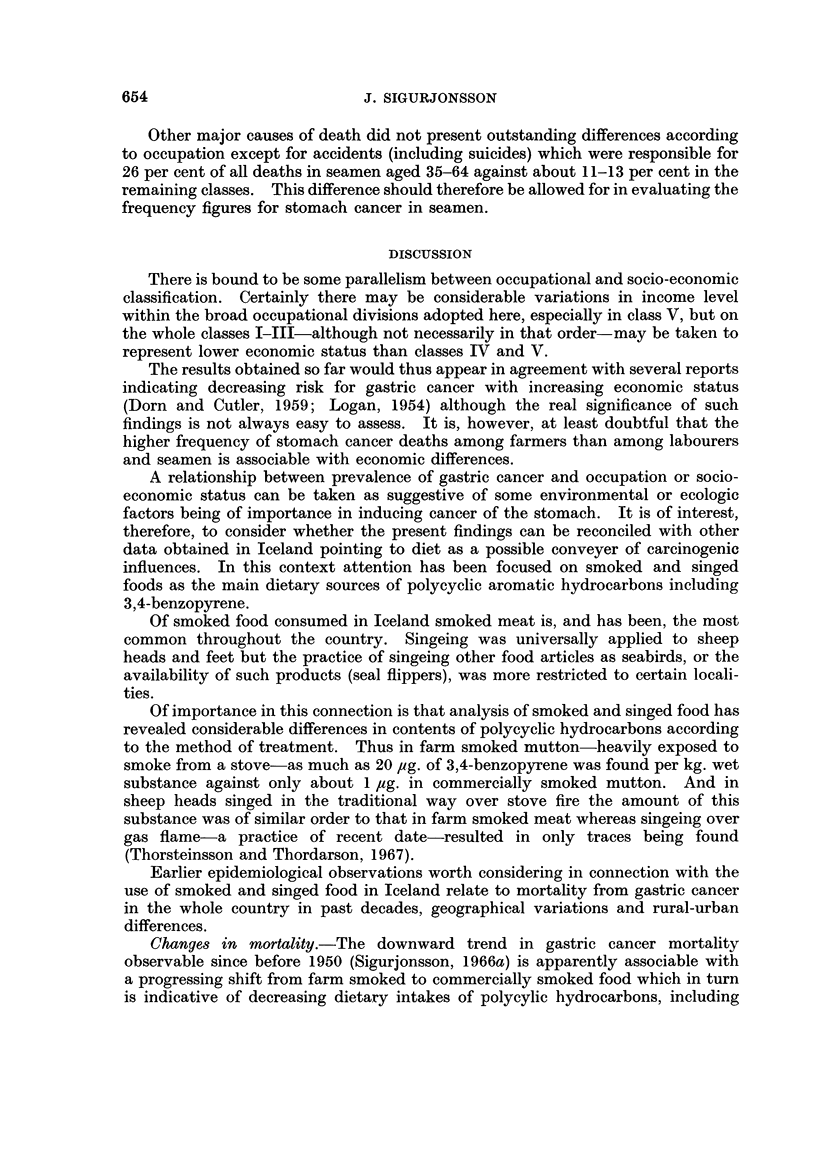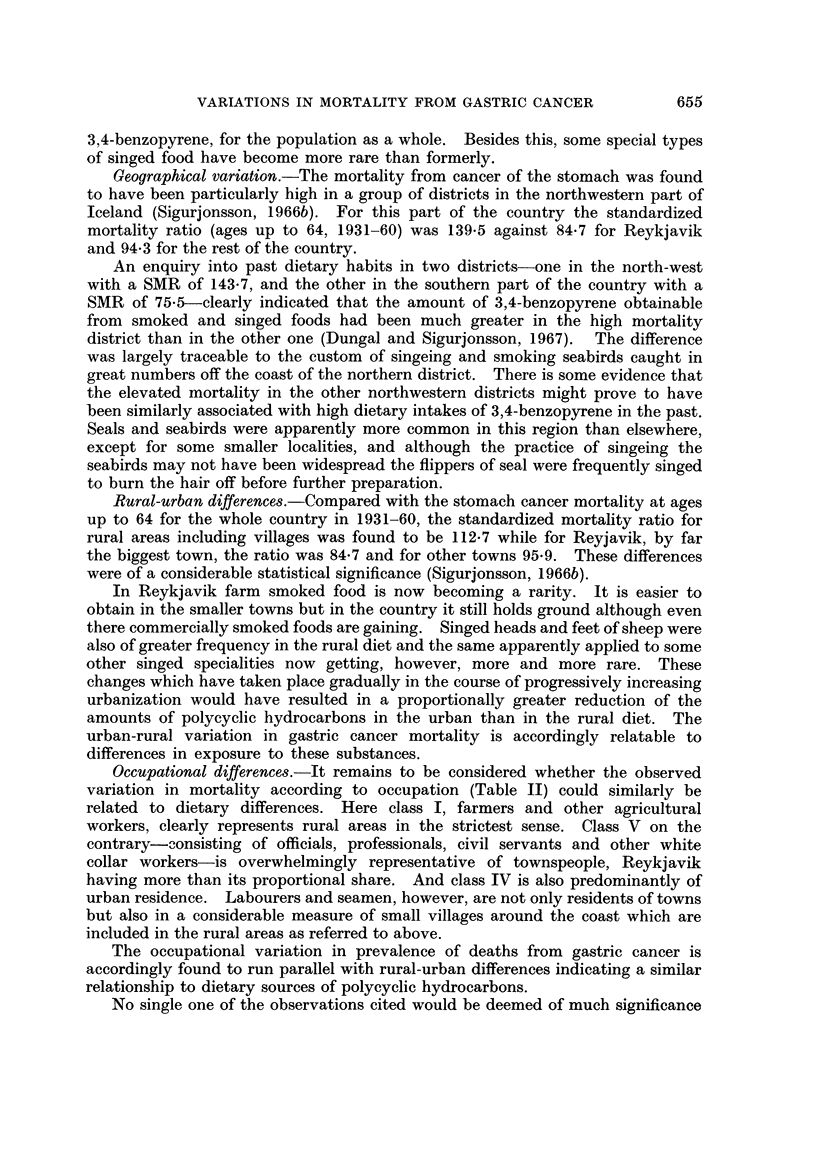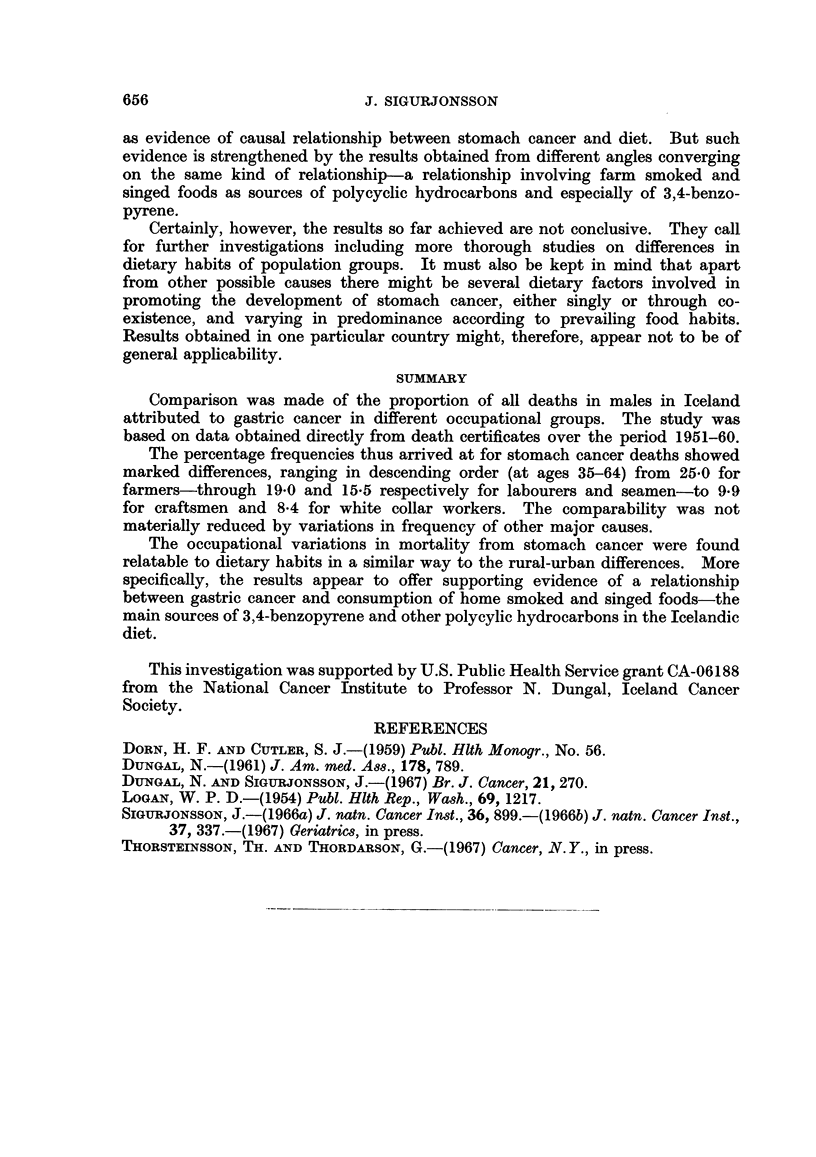# Occupational variations in mortality from gastric cancer in relation to dietary differences.

**DOI:** 10.1038/bjc.1967.76

**Published:** 1967-12

**Authors:** J. Sigurjonsson


### OCCUPATIONAL VARIATIONS IN MORTALITY FROM GASTRIC CANCER IN RELATION TO DIETARY DIFFERENCES

J. SIGURJONSSON

*From the Department of Hygiene, University of Iceland, Reykjavik, Iceland*

Received for publication August 29, 1967.

THE epidemiological pattern of gastric cancer, involving geographical variations and frequently marked downward trends in mortality, is in many ways of considerable interest. Inevitably this variability raises the question of a relationship to diet, especially as other sources of exogenous influences do not appear more likely to furnish an aetiological clue.

In fact, it has long been suspected that some dietary factors might tend to further the development of stomach cancer although results of international comparisons in search for supporting evidence have been elusive. The composition of the human diet is, however, in general a very complex one and it is not always easy to obtain satisfactory information on past dietary habits. Besides that, in dealing with inter-country comparisons, basic dissimilarities between national food customs may make it particularly difficult to single out the most promising factors for further scrutiny, so much the more as not only food constituents as such but also substances generated or taken up during treatment would come into question.

In some respects, therefore, comparative studies conducted within single countries or territories where the general dietary pattern does not vary too much may be easier to evaluate.

The unusually high mortality from gastric cancer in Iceland led Dungal (1961) to suggest an association with the widespread consumption of smoked food, especially smoked meat, in this country. Controlled epidemiological investigations, initiated to look further into this possibility, revealed some marked geographical differences in stomach cancer mortality as well as a certain relationship to population density (Sigurjonsson, 1966*b*), and for the country as a whole a downward trend was clearly demonstrable (Sigurjonsson, 1966*a*).

The present study was undertaken to see whether a frequency pattern for stomach cancer according to occupation could be brought in line with the earlier epidemiological findings in indicating a certain relationship to diet.

### MATERIAL AND METHODS

Death certificates for all adult males dying in Iceland in the period 1951–60 were examined, note being taken of recorded occupation, cause of death, age and residence. Since the material thus obtained was not big enough to allow detailed grouping according to employment, the classification was limited to five broad categories as follows: I, farmers and other agricultural workers, II, labourers, III, seamen, IV, craftsmen and skilled workers, and V, white collar workers.

The agricultural class consists predominantly of farmers, farmhands being in minority. Seamen include fishermen on all kinds of fishing vessels, from small open boats up to trawlers, as well as the crews of the merchant fleet. Classes II and IV, labourers and craftsmen, are fairly well defined except that the definition of skilled worker under class IV may not always have been quite clear. Class V, the white collar workers, is in many ways the most heterogeneous one. This class includes officials, professionals, civil servants, clerical personnel, and those engaged in trading at all levels.

The conformity of occupational classification according to the death certificates to that in the census report for 1950 where also the age distribution is given was esteemed too uncertain in several aspects to rely on in calculating standard mortality rates or ratios. Anyhow, for that purpose the corresponding data for the census year 1960—not yet available—would also have been needed. Instead, therefore, comparison of relative frequencies, i.e. percentages of all deaths attributed to stomach cancer in the various occupation groups, was resorted to.

### RESULTS

Table I shows the number of deaths from gastric cancer by age and occupation and their percentages of all deaths. Up to 65 years of age the proportional

TABLE I.—*Proportion of Gastric Cancer Deaths for Males in Different Occupation Classes. (The Number of Deaths Within Brackets). Iceland,* 1951–60

| Age group | Percentages of all deaths attributed to gastric cancer | | | | | |
|---|---|---|---|---|---|---|
| | All males | I Farmers | II Labourers | III Seamen | IV Craftsmen | V White collar workers |
| 35–44 . | 5·4 | 13·6 (3) | 6·3 (4) | 11·1 (5) | 2·4 (1) | 3·5 (2) |
| 45–54 . | 13·7 | 23·7 (14) | 20·5 (18) | 12·7 (8) | 10·3 (8) | 8·2 (10) |
| 55–64 . | 17·6 | 27·0 (43) | 22·4 (44) | 19·8 (17) | 12·4 (13) | 10·2 (17) |
| 65–74 . | 13·9 | 17·2 (55) | 16·9 (45) | 15·3 (13) | 9·2 (13) | 11·4 (23) |
| 75– . | 6·9 | 7·8 (58) | 8·5 (34) | 7·1 (13) | 5·8 (11) | 5·6 (13) |

figures for stomach cancer deaths are higher in the agricultural class than in any other. They are still above average in the oldest age groups although no longer outranking those for classes II and III. Craftsmen and the white collar group present throughout markedly low values.

Old age mortality statistics for specific causes tend in general to be of reduced reliability (Sigurjonsson, 1967). Thus, for stomach cancer, earlier observations pointed to an increasing underrecording in old age particularly in rural districts (Sigurjonsson, 1966*a* and *b*). Moreover, the class distinctions for the present purposes—especially between the first three classes—is rendered less clear in the older age groups by continuous migration of farmers past middle age to towns and villages where they take up an other employment usually falling either within occupational class II or III. For these reasons the data relating to old ages may be regarded as being of questionable value for comparison.

In Table II, therefore, the old age groups have been left out, leaving ages 35–64 which for simplicity are combined into one group. As could be inferred from Table I the frequency of deaths from gastric cancer at these ages is distinctly highest in the agricultural class, amounting to 25 per cent of all deaths. Next in

TALE II.—*Proportion of Cancer Deaths Among Males Aged* 35–64 *in Different Occupation Classes.* (*Number of Deaths Within Brackets*) *Iceland,* 1951–60

| | Gastric cancer | | Other digestive cancer | All other cancer | Gastric cancer |
|---|---|---|---|---|---|
| Occupational classes | Per cent of all deaths 1 | Per cent of all cancers 2 | Per cent of all deaths 3 | Per cent of all deaths 4 | Per cent of all deaths except B 26* 5 |
| I. Farmers | 25·0 (60) | 56·6 | 3·8 (9) | 15·4 (37) | 27·9 |
| II. Labourers | 19·0 (66) | 52·8 | 4·0 (14) | 13·0 (45) | 23·1 |
| III. Seamen | 15·5 (30) | 53·6 | 2·1 (4) | 11·3 (22) | 18·3 |
| IV· Craftsmen | 9·9 (22) | 37·9 | 4·5 (10) | 11·7 (26) | 12·9 |
| V. White collar workers | 8·4 (29) | 31·9 | 5·8 (20) | 12·2 (42) | 12·1 |
| All males 35–64 years | 14·1 | 46·7 | 3·9 | 12·1 | 17·6 |

* Arteriosclerotic and degenerative heart diseases, No. 420–422.

order are labourers with a percentage of 19 and the figure for seamen would be in the same range if account were taken of their excess mortality from accidents as will be referred to later. Classes IV and V on the other hand show remarkably low frequencies. Taking the figures at their face values the difference between the highest and the lowest levels is found to be of considerable statistical significance as can be ascertained from the data given in the table.

Supporting evidence of the significance of these findings may be seen in the similar variation of the ratio between frequency of stomach cancer and cancer of all sites, as shown in column 2 of Table II, with the highest value or 56·6 per cent in the agricultural group against 37·9 and 31·9 per cent respectively in classes IV and V.

It is noteworthy that class V with the lowest frequency of gastric cancer has highest percentage for other digestive cancer. But in general class differences in frequency of digestive cancer apart from stomach, and of all other cancers, do not appear of much significance.

In dealing with relative frequencies of a specified cause of death as in the present study, the possibility should be considered of an observed variation having emerged mainly as a counterbalancing effect of real differences in prevalence of some other major cause of death. On further examination it was in fact revealed that deaths from arteriosclerotic and degenerative heart diseases (B 26. No. 420–422*) displayed frequency variation according to occupational classes in an inverse order to that for stomach cancer.

Thus in the agricultural class the relative frequency of deaths caused by this disease group was about 10 per cent whereas in the white collar class it was 30 per cent of all deaths occurring at ages 35–64. However, when the effect of this variable was eliminated by calculating the comparative values for stomach cancer as percentages of all deaths other than those due to ischaemic heart diseases (IHD, B26), the pattern remained essentially similar (Table II, column 5). And although the range was not quite as wide it was still significant judged from the figures as such.

*International Classification of Diseases, 1955 Revision.

Other major causes of death did not present outstanding differences according to occupation except for accidents (including suicides) which were responsible for 26 per cent of all deaths in seamen aged 35–64 against about 11–13 per cent in the remaining classes. This difference should therefore be allowed for in evaluating the frequency figures for stomach cancer in seamen.

### DISCUSSION

There is bound to be some parallelism between occupational and socio-economic classification. Certainly there may be considerable variations in income level within the broad occupational divisions adopted here, especially in class V, but on the whole classes I–III—although not necessarily in that order—may be taken to represent lower economic status than classes IV and V.

The results obtained so far would thus appear in agreement with several reports indicating decreasing risk for gastric cancer with increasing economic status (Dorn and Cutler, 1959; Logan, 1954) although the real significance of such findings is not always easy to assess. It is, however, at least doubtful that the higher frequency of stomach cancer deaths among farmers than among labourers and seamen is associable with economic differences.

A relationship between prevalence of gastric cancer and occupation or socio-economic status can be taken as suggestive of some environmental or ecologic factors being of importance in inducing cancer of the stomach. It is of interest, therefore, to consider whether the present findings can be reconciled with other data obtained in Iceland pointing to diet as a possible conveyer of carcinogenic influences. In this context attention has been focused on smoked and singed foods as the main dietary sources of polycyclic aromatic hydrocarbons including 3,4-benzopyrene.

Of smoked food consumed in Iceland smoked meat is, and has been, the most common throughout the country. Singeing was universally applied to sheep heads and feet but the practice of singeing other food articles as seabirds, or the availability of such products (seal flippers), was more restricted to certain localities.

Of importance in this connection is that analysis of smoked and singed food has revealed considerable differences in contents of polycyclic hydrocarbons according to the method of treatment. Thus in farm smoked mutton—heavily exposed to smoke from a stove—as much as 20 $\mu$g. of 3,4-benzopyrene was found per kg. wet substance against only about 1 $\mu$g. in commercially smoked mutton. And in sheep heads singed in the traditional way over stove fire the amount of this substance was of similar order to that in farm smoked meat whereas singeing over gas flame—a practice of recent date—resulted in only traces being found (Thorsteinsson and Thordarson, 1967).

Earlier epidemiological observations worth considering in connection with the use of smoked and singed food in Iceland relate to mortality from gastric cancer in the whole country in past decades, geographical variations and rural-urban differences.

*Changes in mortality.*—The downward trend in gastric cancer mortality observable since before 1950 (Sigurjonsson, 1966*a*) is apparently associable with a progressing shift from farm smoked to commercially smoked food which in turn is indicative of decreasing dietary intakes of polycylic hydrocarbons, including

3,4-benzopyrene, for the population as a whole. Besides this, some special types of singed food have become more rare than formerly.

*Geographical variation.*—The mortality from cancer of the stomach was found to have been particularly high in a group of districts in the northwestern part of Iceland (Sigurjonsson, 1966*b*). For this part of the country the standardized mortality ratio (ages up to 64, 1931–60) was 139·5 against 84·7 for Reykjavik and 94·3 for the rest of the country.

An enquiry into past dietary habits in two districts—one in the north-west with a SMR of 143·7, and the other in the southern part of the country with a SMR of 75·5—clearly indicated that the amount of 3,4-benzopyrene obtainable from smoked and singed foods had been much greater in the high mortality district than in the other one (Dungal and Sigurjonsson, 1967). The difference was largely traceable to the custom of singeing and smoking seabirds caught in great numbers off the coast of the northern district. There is some evidence that the elevated mortality in the other northwestern districts might prove to have been similarly associated with high dietary intakes of 3,4-benzopyrene in the past. Seals and seabirds were apparently more common in this region than elsewhere, except for some smaller localities, and although the practice of singeing the seabirds may not have been widespread the flippers of seal were frequently singed to burn the hair off before further preparation.

*Rural-urban differences.*—Compared with the stomach cancer mortality at ages up to 64 for the whole country in 1931–60, the standardized mortality ratio for rural areas including villages was found to be 112·7 while for Reyjavik, by far the biggest town, the ratio was 84·7 and for other towns 95·9. These differences were of a considerable statistical significance (Sigurjonsson, 1966*b*).

In Reykjavik farm smoked food is now becoming a rarity. It is easier to obtain in the smaller towns but in the country it still holds ground although even there commercially smoked foods are gaining. Singed heads and feet of sheep were also of greater frequency in the rural diet and the same apparently applied to some other singed specialities now getting, however, more and more rare. These changes which have taken place gradually in the course of progressively increasing urbanization would have resulted in a proportionally greater reduction of the amounts of polycyclic hydrocarbons in the urban than in the rural diet. The urban-rural variation in gastric cancer mortality is accordingly relatable to differences in exposure to these substances.

*Occupational differences.*—It remains to be considered whether the observed variation in mortality according to occupation (Table II) could similarly be related to dietary differences. Here class I, farmers and other agricultural workers, clearly represents rural areas in the strictest sense. Class V on the contrary—consisting of officials, professionals, civil servants and other white collar workers—is overwhelmingly representative of townspeople, Reykjavik having more than its proportional share. And class IV is also predominantly of urban residence. Labourers and seamen, however, are not only residents of towns but also in a considerable measure of small villages around the coast which are included in the rural areas as referred to above.

The occupational variation in prevalence of deaths from gastric cancer is accordingly found to run parallel with rural-urban differences indicating a similar relationship to dietary sources of polycyclic hydrocarbons.

No single one of the observations cited would be deemed of much significance as evidence of causal relationship between stomach cancer and diet. But such evidence is strengthened by the results obtained from different angles converging on the same kind of relationship—a relationship involving farm smoked and singed foods as sources of polycyclic hydrocarbons and especially of 3,4-benzopyrene.

Certainly, however, the results so far achieved are not conclusive. They call for further investigations including more thorough studies on differences in dietary habits of population groups. It must also be kept in mind that apart from other possible causes there might be several dietary factors involved in promoting the development of stomach cancer, either singly or through co-existence, and varying in predominance according to prevailing food habits. Results obtained in one particular country might, therefore, appear not to be of general applicability.

SUMMARY

Comparison was made of the proportion of all deaths in males in Iceland attributed to gastric cancer in different occupational groups. The study was based on data obtained directly from death certificates over the period 1951–60.

The percentage frequencies thus arrived at for stomach cancer deaths showed marked differences, ranging in descending order (at ages 35–64) from 25·0 for farmers—through 19·0 and 15·5 respectively for labourers and seamen—to 9·9 for craftsmen and 8·4 for white collar workers. The comparability was not materially reduced by variations in frequency of other major causes.

The occupational variations in mortality from stomach cancer were found relatable to dietary habits in a similar way to the rural-urban differences. More specifically, the results appear to offer supporting evidence of a relationship between gastric cancer and consumption of home smoked and singed foods—the main sources of 3,4-benzopyrene and other polycylic hydrocarbons in the Icelandic diet.

This investigation was supported by U.S. Public Health Service grant CA-06188 from the National Cancer Institute to Professor N. Dungal, Iceland Cancer Society.

### REFERENCES

Dorn, H. F. and Cutler, S. J.—(1959) *Publ. Hlth Monogr.*, No. 56.
Dungal, N.—(1961) *J. Am. med. Ass.*, **178**, 789.
Dungal, N. and Sigurjonsson, J.—(1967) *Br. J. Cancer*, **21**, 270.
Logan, W. P. D.—(1954) *Publ. Hlth Rep., Wash.*, **69**, 1217.
Sigurjonsson, J.—(1966*a*) *J. natn. Cancer Inst.*, **36**, 899.—(1966*b*) *J. natn. Cancer Inst.*, **37**, 337.—(1967) *Geriatrics*, in press.
Thorsteinsson, Th. and Thordarson, G.—(1967) *Cancer, N.Y.*, in press.